# The Role of Myeloid-Derived Suppressor Cells in Multiple Sclerosis and Its Animal Model

**DOI:** 10.14336/AD.2023.0323-1

**Published:** 2024-05-07

**Authors:** Qianling Jiang, Jielin Duan, Luc Van Kaer, Guan Yang

**Affiliations:** ^1^Department of Infectious Diseases and Public Health, Jockey Club College of Veterinary Medicine and Life Sciences, City University of Hong Kong, Kowloon, Hong Kong, China.; ^2^Department of Allergy and Clinical Immunology, State Key Laboratory of Respiratory Disease, National Clinical Research Center for Respiratory Disease, Guangzhou Institute of Respiratory Health, the First Affiliated Hospital of Guangzhou Medical University, Guangzhou, China.; ^3^Department of Pathology, Microbiology and Immunology, Vanderbilt University Medical Center, Nashville, TN 37232, USA.

**Keywords:** multiple sclerosis, experimental autoimmune encephalomyelitis, myeloid-derived suppressor cells, immunotherapy

## Abstract

Myeloid-derived suppressor cells (MDSCs), a heterogeneous cell population that consists of mostly immature myeloid cells, are immunoregulatory cells mainly characterized by their suppressive functions. Emerging findings have revealed the involvement of MDSCs in multiple sclerosis (MS) and its animal model experimental autoimmune encephalomyelitis (EAE). MS is an autoimmune and degenerative disease of the central nervous system characterized by demyelination, axon loss, and inflammation. Studies have reported accumulation of MDSCs in inflamed tissues and lymphoid organs of MS patients and EAE mice, and these cells display dual functions in EAE. However, the contribution of MDSCs to MS/EAE pathogenesis remains unclear. This review aims to summarize our current understanding of MDSC subsets and their possible roles in MS/EAE pathogenesis. We also discuss the potential utility and associated obstacles in employing MDSCs as biomarkers and cell-based therapies for MS.

## 1. Introduction

Multiple sclerosis (MS) is a disease of the central nervous system (CNS) characterized by immune-mediated demyelination, which affects an estimated 2-3 million people worldwide [1]. The exact cause of MS remains unknown, but it is believed to be the result of a combination of genetic and environmental factors [2]. MS is driven by a complex interplay of immune components, which induce CNS inflammation and cause progressive neuronal damage [3, 4]. The most frequently observed symptoms of MS include fatigue, numbness, loss of coordination, vision loss, dizziness, pain, cognitive impairment, depression, as well as bladder and bowel dysfunction [5]. Experimental autoimmune encephalo-myelitis (EAE) is a widely used animal model of MS that resembles MS in many aspects and thus has been instrumental in advancing our understanding of MS. EAE is induced by the injection of myelin antigens such as myelin oligodendrocyte glycoprotein (MOG) or by the adoptive transfer of encephalitogenic T cells, which leads to the activation of immune cells and the infiltration to the CNS [6].

Myeloid-derived suppressor cells (MDSCs) are a heterogeneous population of myeloid lineage cells with potent immunosuppressive properties [7]. Growing evidence suggests that MDSCs modulate immune responses and serve as a key regulator in the generation and perpetuation of autoimmune diseases [8]. Owing to their prominent suppressive effects on T cell function in cancer development [7], increasing studies have focused on their role in MS/EAE, in which T cells are the main culprit. Recent studies have shown that MDSCs accumulate in the peripheral blood of MS patients and various organs of EAE mice, suggesting a potential role for MDSCs in the pathogenesis of these diseases. Since MS patients display an unpredictable disease course with various clinical manifestations, predicting the possible disease course of MS patients and their potential responses to therapies via biological markers is crucial. This review provides an overview of the aberrant changes and pleiotropic functions of MDSCs in MS and EAE, elucidating their potential utility as both biomarkers and therapeutic targets for MS.

## 2. Myeloid-derived suppressor cells

MDSCs are a highly plastic and heterogeneous population predominantly consisting of immunosuppressive immature myeloid cells (IMCs) that are stimulated and expanded in the context of multiple pathological conditions such as cancer, chronic inflammation, infection, and traumatic stress [9, 10]. Most of our understanding of MDSCs has been derived from cancer studies, where these cells are identified as immunosuppressive cells with the capacity to promote cancer cell proliferation and survival [11]. More recently, interest in the roles of these cells in autoimmune diseases has increased dramatically. In this section, we will briefly discuss these MDSC subsets.

**Figure 1.**
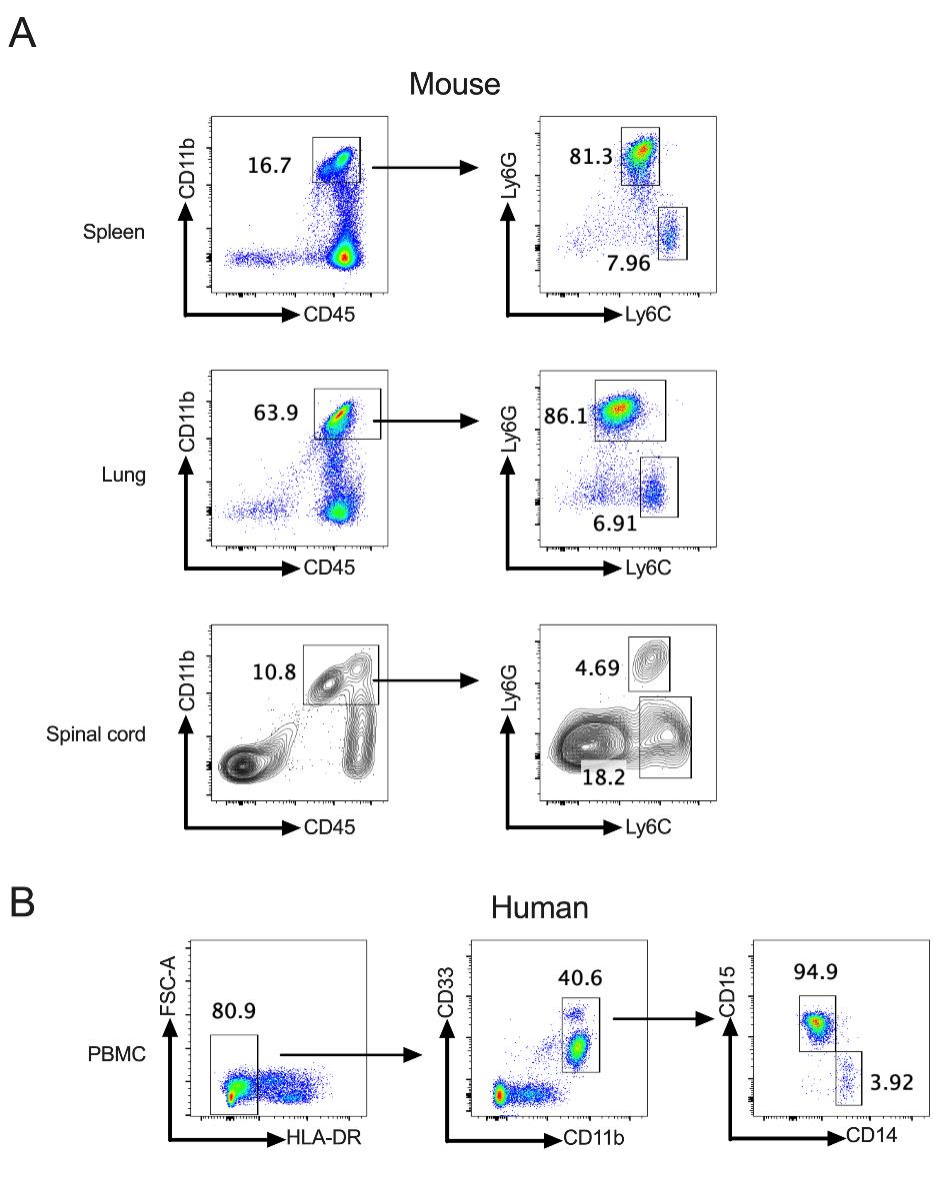
**Gating strategies for the identification of mouse/human MDSC subsets**. (**A**) Gating strategies used to define MDSCs subpopulation in spleen, lung, and spinal cord of C57BL/6 EAE mice. CD45^+^CD11b^+^ cells were gated, and the proportion of Ly6C^+^ and Ly6G^+^ cells was evaluated. M-MDSCs are characterized as CD45^+^CD11b^+^Ly6G^-^Ly6C^high^, and PMN-MDSCs are defined as CD45^+^CD11b^+^ Ly6G^+^Ly6C^low^. (**B**) Gating strategies for the identification of human MDSC subsets. Gating strategies used to define MDSC subpopulations in the peripheral blood of a human. M-MDSCs are defined as CD11b^+^HLA-DR^-/low^ CD33^+^ CD14^+^CD15^-^, and PMN-MDSCs are defined as CD11b^+^CD33^mid^CD14^-^CD15^+^. This gating strategy is partly adopted from [13].

MDSCs consist of two principal subsets based on their origins from the granulocytic or monocytic myeloid lineages: polymorphonuclear/granulocytic MDSCs (PMN-MDSCs/G-MDSCs) and monocytic MDSCs (M-MDSCs) [12]. PMN-MDSCs originate from common myeloid progenitors, as well as granulocytic precursors and monocytic-like precursors, while M-MDSCs are derived from common myeloid progenitors and monocytic precursors [12]. As PMN-MDSCs and M-MDSCs are phenotypically and morphologically similar to classical neutrophils and monocytes, phenotypic criteria alone are insufficient to differentiate these subsets. Therefore, some cell surface markers have been employed to complement the definitions of MDSC subsets. In humans, M-MDSCs are defined as CD11b^+^CD14^+^HLA-DR^-/low^CD15^-^, and PMN-MDSCs are defined as CD11b^+^CD14^-^CD15^+^ or CD11b^+^CD14^-^CD66b^+^ [13]. Of note, the expression level of CD33, a general myeloid marker, was also used to differentiate between human M-MDSCs (CD33^+^) and PMN-MDSCs (CD33^mid^) (Fig. 1) [13]. Although monocytes in human peripheral blood display a similar phenotype to M-MDSCs, they can be distinguished from M-MDSCs by the expression of major histocompatibility complex class II molecules - HLA-DR [12, 14]. To separate neutrophils from PMN-MDSCs in human PBMC, standard ficoll density gradient centrifugation is usually employed since PMN-MDSCs are found in the low-density fraction [13]. Furthermore, human PMN-MDSCs specifically express the lectin-like oxidized low-density lipoprotein receptor 1 (LOX1) [15], which could be utilized to differentiate them from classic neutrophils. Additionally, CXC-chemokine receptor 1, which is typically associated with neutrophil functions, is upregulated in human M-MDSCs, indicating its potential as a novel marker for M-MDSCs [16].

In mice, MDSCs are generally defined as CD11b^+^Gr-1^+^, with M-MDSCs characterized as CD11b^+^Ly6G^-^Ly6C^high^, and PMN-MDSCs characterized as CD11b^+^ Ly6G^+^Ly6C^low^ (Fig. 1) [13]. It remains challenging to distinguish PMN-MDSCs and M-MDSCs from neutrophils and monocytes in mice based on cell surface markers [12]. Recent studies have identified CD84 as a promising marker for defining human and murine MDSCs in cancer [17]. However, it is yet to be determined whether CD84-defined MDSCs are breast cancer-specific [12]. In addition, human and murine MDSCs can also be distinguished from mature neutrophils and monocytes by their specific genomic, proteomic, and metabolic profiles, functional activities, and biochemical characteristics [18]. For example, a major characteristic of MDSCs is the high expression of arginase-1 (Arg-1) and corresponding arginase activity [12]. Apart from these two major subgroups, additional MDSC populations have been recently identified. In humans, early-stage MDSCs, comprising more immature progenitors which subsequently differentiate into PMN-MDSCs or M-MDSCs, are defined as CD33^+^HLA-DR^-^Lin^-^ (Lin: CD3, CD14, CD15, CD19, and CD56) [13]. However, the characterization of early-stage MDSCs in mice remains unsolved. Another new subpopulation, Eo-MDSCs that phenotypically resemble immature eosinophils, has been identified in mice infected with *Staphylococcus aureus* [19].

In healthy individuals, myelopoiesis is a highly regulated and coordinated process during which hematopoietic stem cells develop into IMCs and quickly differentiate into various mature myeloid cells such as granulocytes, macrophages, dendritic cells, and monocytes in the bone marrow. Under normal conditions, only a minute proportion of IMCs migrate from bone marrow to the peripheral tissues and the occurrence of MDSCs in the body is typically quite low. One exception occurs during pregnancy, immunosuppressive IMCs (MDSCs) expand in the umbilical cord blood, contributing to maternal-fetal tolerance through arginase-mediated suppression of maternal T cell responses [20-22]. In pathological conditions, including cancer, autoimmune diseases, sepsis, trauma, and infections, MDSCs accumulate in various organs [23].

In healthy mice, the bone marrow contains a higher portion of CD11b^+^Gr1^+^ cells (>20% of total cells) compared with other tissues [7]. In healthy humans, MDSCs make up 0.5% of the total peripheral blood immune cells [24]. Interestingly, PMN-MDSCs are enriched in human breast milk, where their prevalence is 20-fold higher than in peripheral blood, whereas the levels of M-MDSCs in breast milk are lower than in peripheral blood [25]. However, accurate frequencies and numbers of MDSCs in other tissues of healthy human subjects remain unclear due to limited sample size or inconsistent data [26].

The primary function of MDSCs in immune suppression is achieved through several mechanisms. In general, M-MDSCs preferentially utilize mechanisms associated with the production of nitric oxide (NO), immunosuppressive cytokines including IL-10 and TGF-β, and the expression of immune regulatory molecules such as programmed death-ligand-1 (PD-L1); whereas PMN-MDSCs exert their effects mainly through the production of reactive oxygen species (ROS), peroxynitrite, Arg-1, and prostaglandin E_2_ [14]. L-arginine is metabolized into NO and citrulline by inducible nitric oxide synthase (iNOS), and Arg-1 catalyzes L-arginine into ornithine and urea. Arg-1 and iNOS produced by MDSCs lead to depletion of L-arginine, and the reduced level of L-arginine inhibits T cell proliferation by lowering the expression of the CD3ζ chain and cell cycle regulators cyclin D3 and cyclin-dependent kinase 4 [27, 28]. Meanwhile, NO suppresses T cell activities by inhibiting Janus kinase and (signal transducer and activator of transcription 3 (STAT3) signaling, decreasing the expression of major histocompatibility complex class II molecules expressed on antigen-presenting cells, and inducing T cell apoptosis [29]. ROS, such as hydrogen peroxide generated by NADPH oxidase, inhibits the differentiation of IMCs into macrophages and dendritic cells [30]. ROS-related peroxynitrite, a powerful oxidant, induces T cell apoptosis by blocking tyrosine phosphorylation of proteins needed for T cell activation or blocks T-cell migration by nitrating T-cell-specific chemokines [31, 32].

MDSCs play a key role in mediating immune cell functions, although with differential outcomes in distinct experimental systems. For example, MDSCs have been reported to induce the development of regulatory T cells (Tregs) in the presence of IFN-γ and IL-10 [33], whereas other studies have reported no correlation between Tregs and MDSCs during tumor growth [34], or even to partially suppress Treg expansion in a rat kidney allograft tolerance model [35]. In addition, MDSCs inhibited natural killer cell killing function through membrane-bound TGF-β1 in tumor-bearing models [36], whereas another study reported that MDSCs activate natural killer cells in tumor-bearing mice [37]. These conflicting findings might be due to the heterogeneity of MDSCs.

The deleterious role of MDSCs in tumors is well-established, whereas only limited studies have explored the roles and functions of MDSCs in autoimmune diseases. These limited studies have revealed the accumulation of MDSCs in the inflamed tissues and lymphoid organs of patients with rheumatoid arthritis (RA), type 1 diabetes, systemic lupus erythematosus (SLE), inflammatory bowel disease, and MS [reviewed in [8]]. Attention to the role of MDSCs in autoimmune diseases has been aroused in recent years. Interestingly, it has been shown that MDSCs have both beneficial and pathogenic roles in autoimmune diseases, contributed by their immunosuppressive and proinflammatory functions, respectively. The following sections focus on the latest studies concerning the role of MDSCs in MS and EAE. Apart from these studies focusing on MDSCs, there are relevant reports on MS/EAE concerning immature Ly6C^high^ and Ly6G^+^ myeloid cells that are not described as “MDSCs” but likely represent M-MDSCs and PMN-MDSCs based on their surface characterization, and we will also include them in this review.

## 3. MDSCs in MS

### 3.1 M-MDSCs in MS

A recent study has revealed a phenotypic and functional shift of MDSCs in peripheral blood mononuclear cells (PBMCs) during MS progression. This study showed higher frequencies of M-MDSCs in relapsing-remitting MS (RRMS) patients naïve to disease-modifying therapies (DMTs) during the relapse stage as compared with the remission stage and healthy controls [38]. Another study similarly found an increased frequency of M-MDSCs in the blood of newly diagnosed RRMS patients but did not describe whether the peripheral blood was collected from the relapse or remission stage [39]. Although not significant, RRMS patients in remission appeared to contain lower frequencies of M-MDSCs compared to healthy controls [38], which might help explain the decreased numbers and frequencies of M-MDSCs in RRMS patients observed in another study with most samples derived from patients with RRMS during remission [40]. However, two studies reported no change of M-MDSCs among PBMCs between RRMS patients and healthy individuals [41, 42]. In addition, frequencies of M-MDSCs were lower in patients with secondary progressive MS (SPMS) compared to healthy controls [38]. Notably, M-MDSCs from SPMS patients expressed lower levels of CD86 (co-stimulatory molecule), CD163 (myeloid cell marker), and mRNA for *IL10* and *HMOX1* compared to those from RRMS patients, suggesting possible impaired suppressive capacity, which was confirmed by the capacity of these cells to mediate *in vitro* T cell expansion rather than T cell suppression [38]. However, this study did not clarify whether these SPMS patients had received DMTs, which may contribute to these alterations. Interestingly, a recent study has shown a positive correlation between M-MDSC abundance at baseline and improved responses to fingolimod treatment in MS patients, suggesting that M-MDSCs have the potential to serve as predictive biomarkers for assessing the efficacy and responsiveness of fingolimod treatment in MS [43]. These observations suggest that the frequency of M-MDSCs is modulated during MS progression and drug treatments, indicating their possible use as biomarkers for predicting disease course and treatment responses.

### 3.2 PMN-MDSCs in MS

Like M-MDSCs, the frequency of PMN-MDSCs in PMBCs from RRMS patients varies. Studies have revealed either no alteration of PMN-MDSCs in PBMCs from RRMS patients [39, 40] or a significant increase of these cells compared to healthy individuals [41, 42]. In addition, conflicting results have been reported regarding the frequency of PMN-MDSCs in PBMCs between the RRMS relapse stage and remission stage [38, 42, 44]. Examination of paired blood samples from MS patients during relapse and at a follow-up visit revealed an increase in the frequency of LOX1^+^ PMN-MDSCs in those who achieved complete control of inflammatory disease activity and met the criteria for “no evidence of disease activity”, whereas no significant changes were observed in those with ongoing disease activity [42]. This intriguing finding suggests a potential role for LOX1^+^ PMN-MDSCs in the maintenance of “no evidence of disease activity” in MS. Moreover, one study has demonstrated that methylprednisolone-mediated MS amelioration is associated with an expansion of PMN-MDSCs, suggesting a possible beneficial role of PMN-MDSCs in MS amelioration or as an indicator of treatment efficacy [41].

Possible explanations for those discrepancies regarding alterations of MDSC subsets in MS include genetic differences and samples obtained from different disease stages, as well as previous immunoregulatory treatments. An additional contributing factor may be the different characterization standards and flow cytometry gating strategies employed (Table 1). For example, one study reported increased levels of M-MDSCs (CD14^+^HLA-DR^-/low^) in the peripheral blood of RRMS patients [39], whereas another study that defined M-MDSCs as CD14^+^CD15^-^CD33^+^HLA-DR^-^ cells reported opposite findings [40]. Meanwhile, one study found no significant difference when they characterized M-MDSCs as CD14^+^CD15^-^HLA-DR^low^ cells [42]. Likewise, regarding PMN-MDSCs, certain studies have neglected the use of LOX1 as a marker, which may result in inconsistent findings [38-41, 44]. As such, there is a need for more consistent characterization of MDSC subsets, with the use of novel markers encouraged to ensure accurate and consistent results. Collectively, these findings suggest the involvement of M-MDSCs and PMN-MDSCs in MS, but whether they directly contribute to MS pathogenesis, or their alterations are by-products of other processes requires further study.

**Table 1:** MDSCs in MS.

Disorder	Sample information	M-MDSCs	Surface marker	PMN-MDSCs	Surface Marker	Findings	Ref.
**RRMS**	Italy: Untreated RRMS (n=52) HC (n=26)	(peripheral blood)↑	CD14^+^ HLA-DR^-/low^	(peripheral blood)→	CD14^-^ CD15^+^ CD33^+^ HLA-DR^-/low^	RRMS patients possessed increased levels of M-MDSCs in their peripheral blood.	[39]
**RRMS**	USA: Untreated RRMS (n=24) GA treated MS (n=10) HC (n=16)	(peripheral blood)↓	CD14^+^ CD15^-^ CD33^+^ HLA-DR^-^	(peripheral blood)→	CD14^-^ CD15^+^ CD33^+^ HLA-DR^-^	RRMS patients possessed decreased levels of M-MDSCs in their peripheral blood.	[40]
**RRMS/CIS**	Germany: RRMS/CIS (n=70) HC (n=31)	(peripheral blood)→	CD14^+^ CD15^-^ HLA-DR^low^	(peripheral blood)↑	CD11b^int^ CD15^+^ CD33^+^ LOX1^+^	LOX1^+^PMN-MDSCs accumulated in the blood of patients with RRMS/CIS. The frequency of PMN-MDSCs was significantly lower in MS patients who recently experienced a relapse compared to stable MS subjects.	[42]
**RRMS**	Greece: Active MS (n = 14) Remission (n = 17) HC (n = 26)			(peripheral blood)↑	CD14^-^ CD15^+^ CD33^+^ HLA-DR^-/low^	The frequency and number of PMN-MDSCs significantly increased in the peripheral blood of patients with active MS compared with patients in remission or healthy controls.	[44]
**RRMS**	China: RRMS (n=30) HC (n=20) Methylprednisolone treated RRMS (n=12)	(peripheral blood)→ After treatment →	CD11b^+^ CD14^+^ CD33^+^ HLA-DR^-^ SSC^low^ CD66b^-^	(peripheral blood)↑ After treatment ↑	CD11b^+,^ CD14^-^ CD33^+^ HLA-DR^-^ SSC^high^ CD66b^+^	Methylprednisolone attenuated MS by inducing PMN-MDSCs via glucocorticoid receptor β signaling and S100A8/9 up-regulation.	[41]
**RRMS**	Sweden: Untreated relapse (n=11) Untreated remission (n=19) HC (n=20)	(peripheral blood)↑	CD11b^+^ CD14^+^ HLA-DR^low^	(peripheral blood)↑	CD11b^+^ CD14^-^ CD33^+^ HLA-DR^low^	The frequency of M-MDSCs was increased in RRMS-relapse compared to RRMS-remission and HC. The frequency of PMN-MDSCs were increased in RRMS-relapse compared to RRMS-remission.	[38]
**SPMS**	Sweden: SPMS (n=19) HC (n=20)	(peripheral blood)↓	CD11b^+^ CD14^+^ HLA-DR^low^	(peripheral blood)↓	CD11b^+^ CD14^-^ CD15^+^ CD33^+^ HLA-DR^low^	SPMS patients showed a decreased frequency of M-MDSCs and PMN-MDSCs compared to healthy controls.	[38]

Abbreviations: CIS: clinically isolated syndrome; HC: healthy control; HLA-DR: human leucocyte antigen DR; LOX-1: lectin-type oxidized LDL receptor 1; MDSCs: myeloid-derived suppressor cells; M-MDSCs: monocytic myeloid-derived suppressor cells; MS: multiple sclerosis; PMN-MDSCs: polymorphonuclear myeloid-derived suppressor cells; RRMS: relapsing-remitting multiple sclerosis; SPMS: secondary progressive multiple sclerosis.

## 4. MDSCs in EAE

Most studies on MDSCs in MS have been performed using the EAE model, providing insights into their likely biological functions and underlying roles in disease pathogenesis. Similar to the studies for MS, the number or frequency of MDSCs changes during EAE progression (Table 2). However, trends in MDSC changes reported in different organs and tissues of EAE mice are more consistent than in MS patients. For example, MDSCs expanded in the spleen and peripheral blood at the early stage of EAE [45], and the levels of MDSCs in the demyelinated spinal cord [46] and spleen [47] correlated with disease severity. In particular, M-MDSCs accumulated in the spleen, blood, CNS, and bone marrow of EAE mice [48], and PMN-MDSCs were enriched in the lung, spleen, CNS, and draining lymph node of EAE mice [44, 49, 50]. One possible explanation for these findings is that studies with experimental mice are more consistent and tractable with regard to genetic variations and disease course compared with individual RRMS patients.

The name MDSCs was coined to reflect their *in vitro* immunosuppressive effects, mainly on T cells, but their *in vivo* activities appear to be much more complex. Interestingly, MDSCs appear to play a dual role in EAE as they are reported to either suppress or enhance EAE under different experimental conditions, and comprehending the underlying mechanisms of this functional switch in MDSCs is crucial for the development of novel immunotherapies for MS.

**Table 2:** MDSCs in EAE.

Disorder	Treatment/ effects	M-MDSCs	Surface marker	PMN-MDSCs	Surface Marker	Findings	Ref.
**EAE**		MDSCs: CD11b^+^Gr-1^+^Arg-1^+^M-CSF1R^+^ ↑(spinal cord)				The density of MDSCs paralleled the clinical score of EAE. FACS-sorted MDSCs from the spinal cord of MOG-immunized mice limited inflammation by promoting T lymphocyte apoptosis.	[46]
**EAE**		MDSCs: Ly6C^high^Ly6G^-/low^ ↑(spleen)				The proportion of splenic MDSCs was related to the severity of the clinical course and tissue damage extent in EAE.	[47]
**EAE**		↑(spleen) ↑(bone marrow) ↑(blood) ↑(CNS)	CD11b^+^ Ly6C^high^ Ly6G^-^			Splenic CD11b^+^Ly-6C^high^ inflammatory monocytes isolated from BALB/c mice at 10 days post-MOG-immunization efficiently suppressed T cell proliferation and induce T cell apoptosis through the production of NO.	[48]
**EAE**			CD11b^low^ Ly6C^high^	↑(lung)	CD11b^high^ Ly6C^low^	PMN-MDSCs accumulated in the lung during EAE. FACS-sorted PMN-MDSCs collected at 7 days post-MOG-immunization promoted Th17 cell differentiation.	[49]
**EAE**			CD11b^low^ Ly6C^high^	↑(lung)	CD11b^high^ Ly6C^low^	Lung MDSCs isolated 10 days post-MOG-immunization suppress CD8^+^ T cell function via iNOS.	[50]
**EAE**		→ (spleen)	CD11b^high^ Ly6C^+^	↑ (spleen) ↑ (spinal cord) ↑ (draining lymph node)	CD11b^high^ Ly6C^-^ Ly6G^+^	PMN-MDSCs accumulated in the CNS and peripheral lymphoid compartments of EAE mice.	[44]
**EAE**	CBD (Amelioration)	↑(spleen) →(CNS) →(mesenteric lymph node)	CD45^+^ CD11b^+^ Ly6C^+^ Ly6G^-^	→(spleen) ↑(CNS) →(mesenteric lymph node)	CD45^+^ CD11b^+^ Ly6C^+^ Ly6G^+^	CBD treatment increased MDSC subsets both in the periphery and in the CNS of EAE mice.	[73]
**EAE**	CBD (Amelioration)	↑(spleen)→	CD11b^+^ Ly6C^+^ Ly6G^-^	↑(spleen)→	CD11b^+^ Ly6C^low^ Ly6G^+^	Oral administration of CBD did not affect the percentage of MDSCs in the spleen.	[76]
**EAE**	CBD (Amelioration)	MDSCs: CD11b^+^ Gr-1^+^ ↑(peritoneal cavity) ↓(CNS)				CBD attenuated EAE through induction of MDSCs in the periphery.	[75]
**EAE**	Serum albumin (SA)-IL-4 fusion protein (Amelioration)	↓(draining lymph nodes)	CD45^+^ CD11b^+^ Ly6C^+^ Ly6G^-^	↑(draining lymph nodes)	CD45^+^ CD11b^+^ Ly6C^+^ Ly6G^+^	MDSCs induced T-cell suppression possibly through the PD-1/PD-L1 axis.	[81]
**EAE**	NAD^+^ (Amelioration)	MDSCs: CD11b^+^Gr-1^+^ ↑(spleen)				NAD^+^ treatment induced the expansion of MDSCs in the spleen and promoted Arg-1 expression in the spleen and spinal cord.	[77]
**EAE**	IFN-β (Amelioration)	↑(spleen)	CD11b^+^ Ly6C^high^ Ly6G^-/low^	→(spleen)	CD11b^+^ Ly6C^int^ Ly6G^high^	IFN-β treatment induced the expansion of M-MDSCs and preserved MDSC immaturity.	[78]
**EAE**	MiR-223 ablation (Amelioration)	↑(CNS) ↑(spleen)	CD11b^+^ Ly6C^high^	→(CNS) ↑(spleen)	CD11b^+^ Gr1^+^	MIR233 regulated the number and function of M-MDSCs in EAE, which was associated with increased Arg-1 and STAT3 expression.	[40]
**EAE**	calpain inhibitor SNJ-1945 (Amelioration)	MDSCs: Gr-1^+^Integrin-a^+^ ↑(lymph nodes)				SNJ-1945 treatment induced the expansion of MDSCs in the LN.	[79]
**EAE**	Inhibition of activated PC (Amelioration)	↑(spleen)	CD11b^+^ Ly6C^high^ Ly6G^-^	↑(spleen)	CD11b^+^ Ly6C^low^ Ly6G^+^	Inhibition of activated PC induced the expansion of splenic MDSCs and increased the expression level of IL-4Ra on MDSCs.	[80]
**EAE**	α-GalCer (Amelioration)	↑(spleen) ↑(CNS)	CD11b^+^ Ly6C^high^	↑(spleen) ↑(CNS)	CD11b^+^ Ly6G^high^	α-GalCer-activated iNKT cells cooperated with MDSCs in the attenuation of EAE.	[60]
**EAE**	Methylprednisolone (Amelioration)	→ (peripheral blood) → (spleen)	CD11b^+^ Gr-1^+^ Ly6C^high^ Ly6G^-^	↑ (peripheral blood) → (spleen)	CD11b^+^ Gr-1^+^ Ly6C^-^ Ly6G^high^	Methylprednisolone did not significantly change the level of M-MDSCs and PMN-MDSCs in PBMCs and spleen, although methylprednisolone alleviated EAE clinical symptoms.	[41]
**EAE**	Gemcitabine (Amelioration)	MDSCs: CD11b^+^Gr-1^+^ ↑(peripheral blood)↓ ↑(spleen)↓				Spleen-derived MDSCs isolated from MOG-immunized mice by MACS promoted Th17 cell differentiation through IL-1β. Selective depletion of MDSCs reduced Th17 cells and ameliorated EAE.	[45]
**EAE**	Type I IFN receptor ablation in T regulatory cells (aggravation)	↓(draining lymph nodes) → (spleen) → (CNS) → (bone marrow)	CD11b^+^ Ly6C^high^ Ly6G^-^	↓(draining lymph nodes) → (spleen) → (CNS) → (bone marrow)	CD11b^+^ Ly6C^-^ Ly6G^+^	Depleting IFNAR in Tregs led to a reduction in MDSCs in dLN.	[53]
**EAE**	Retinoid Am80 (aggravation)	MDSCs: CD11b^+^Ly6C^high^Ly6G^-/low^ ↓(spleen)				AM80 delayed EAE recovery by decreasing the proportion of splenic MDSCs, and T cell density and viability were promoted.	[52]

BLACK: change compared to healthy control. RED: change compared to the vehicle-treated group with EAE. Abbreviations: CBD: cannabidiol; CNS: central nervous system; EAE: experimental autoimmune encephalomyelitis; FACS: Fluorescence-activated cell sorting; MACS: Magnetic-activated cell sorting; MDSCs: myeloid-derived suppressor cells; M-MDSCs: monocytic myeloid-derived suppressor cells; PMN-MDSCs: polymorphonuclear myeloid-derived suppressor cells.

### 4.1 Mechanisms of MDSC-mediated EAE suppression

MDSC-mediated EAE protection is achieved through various mechanisms. Firstly, MDSCs alleviate EAE by inhibiting autoreactive T cell proliferation and inducing T cell anergy and apoptosis. Specifically, CNS M-MDSCs limited inflammation by inducing T cell apoptosis [46]. Splenic PMN-MDSCs also inhibited CD4^+^ T cell proliferation in an Arg-1-mediated cell contact-dependent manner [51]. PMN-MDSCs can suppress T cell function via upregulation of PD-L1. PD-L1 expression was upregulated in PMN-MDSCs from EAE mice, and their adoptive transfer ameliorated EAE by inhibiting autoantigen-specific Th1 and Th17 cell priming in a PD-L1-dependent pathway [44]. In contrast, adoptive transfer of PD-L1-deficient PMN-MDSCs from MOG-immunized mice failed to attenuate EAE and suppress encephalitogenic T cell expansion [44]. Consistently, promoting MDSC differentiation into mature myeloid cells lacking suppressor activities enhanced T cell viability [52].

In addition to these mechanisms, the beneficial effects provided by MDSCs might be mediated via Treg induction. PMN-MDSCs induced Treg expansion *in vitro* [51], suggesting a possible mechanism for the amelioration of EAE. However, the underlying mechanism behind the interaction between MDSCs and Tregs in EAE is less clear, as illustrated by an *in vivo* study that failed to identify significant changes in the frequency of Tregs after adoptive transfer of PMN-MDSCs [44]. Interestingly, one study showed that deletion of IFN alpha receptor on Tregs resulted in failure to recruit MDSCs to draining lymph nodes and EAE enhancement, suggesting a type I interferon-dependent regulatory role of Tregs on MDSC trafficking [53], raising the possibility of a positive immunoregulatory loop between MDSCs and Tregs in EAE.

MDSC-mediated suppression of EAE is potentially achieved by promoting the remyelination process in the CNS, as suggested by the capacity of osteopontin secreted by M-MDSCs to promote the survival, proliferation, and differentiation of oligodendrocyte precursor cells, indicating a possible supportive role of M-MDSCs in the remyelination of EAE mice [54]. However, whether PMN-MDSCs also contribute to the remyelination process remains unclear.

**Figure 2.**
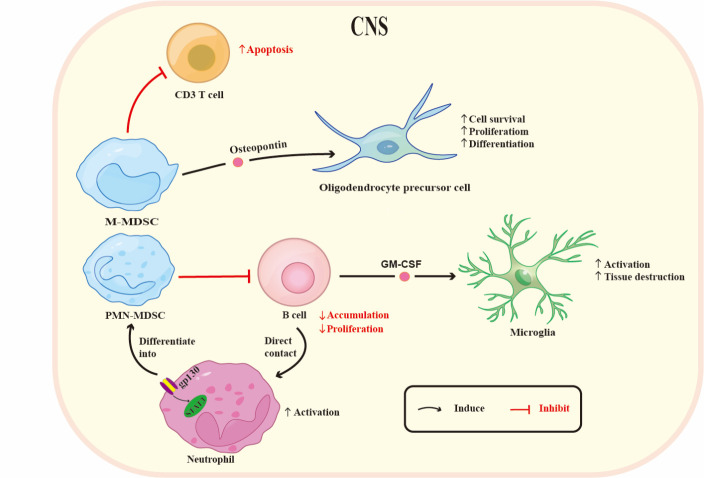
**Proposed MDSC-related immune interactions in the CNS during EAE development**. M-MDSCs in the CNS induce apoptosis of T cells. M-MDSCs promote oligodendrocyte precursor cell survival, proliferation, and differentiation via production of osteopontin. B cells directly contact neutrophils and promote their differentiation into PMN-MDSCs with activation of gp130/STAT3 signaling. In turn, PMN-MDSCs restrain B cell accumulation and block the activation of the tissue-destructive microglia via GM-CSF secretion. Abbreviations: CNS: central nervous system; M-MDSC: monocytic myeloid-derived suppressor cell; PMN-MDSC: polymorphonuclear myeloid-derived suppressor cell; STAT3: signal transducer and activator of transcription 3.

Moreover, MDSCs might attenuate EAE by preventing pathogenic B cell accumulation. Both *in vitro* and *in vivo* studies have revealed that PMN-MDSCs inhibit B cell differentiation and proliferation [55, 56], suppress T follicular helper cell function [57], and induce regulatory B cells as well as IgA^+^ antibody-secreting B cells in different diseases [58, 59]. A recent study on EAE reported an autoregulatory loop among Ly6G^+^ neutrophils, PMN-MDSCs, and B cells in the CNS (Fig. 2). B cells guide neutrophils to differentiate into PMN-MDSCs with the activation of gp130/STAT3 signaling. The PMN-MDSCs then restrain B cell accumulation, which could block the activation of tissue-destructive microglia, thus decreasing disease severity [42]. However, the underlying molecular mechanism by which PMN-MDSCs control B cells in the CNS during EAE needs further study.

Lastly, MDSCs cooperated with glycolipid-activated invariant natural killer T (iNKT) cells to attenuate EAE. Our previous study reported that α-galactosylceramide, the prototypical agonist of iNKT cells, mediated the expansion and immunosuppressive properties of MDSCs in MOG-immunized EAE mice, and that MDSC depletion eliminated the protective effects of iNKT cell activation against EAE [60]. Meanwhile, adoptive transfer of MDSCs derived from α-galactosylceramide-treated mice attenuated EAE in recipient mice. This protection against EAE might be due to the production of cytokines and other factors by iNKT cells (GM-CSF, IL-4, and/or IFN-γ) and MDSCs (iNOS, Arg-1, and/or IL-10) [60].

### 4.2 Mechanisms of MDSC-mediated EAE exacerbation

**Figure 3.**
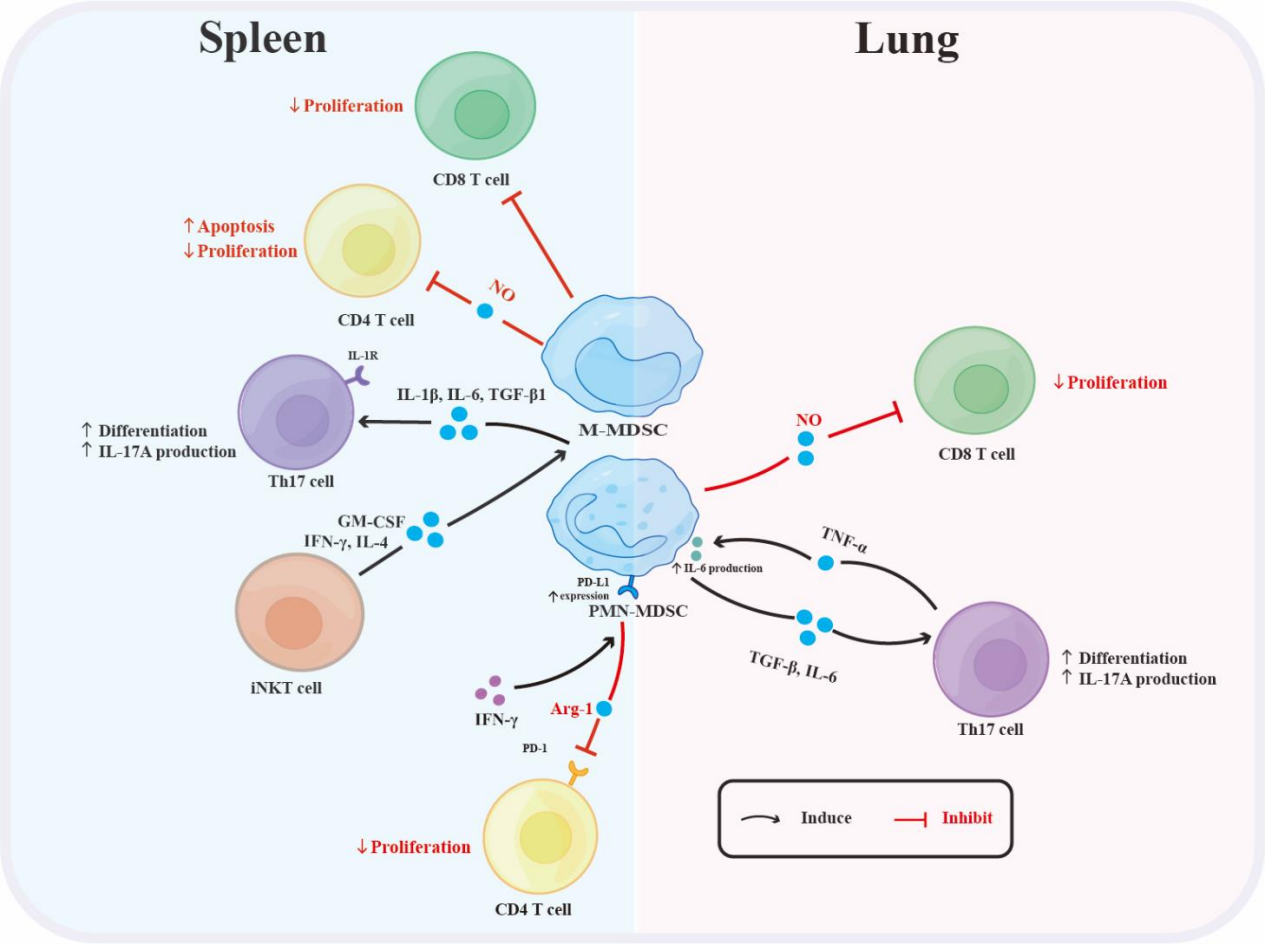
**Proposed MDSC-related immune interactions in the spleen and lung during EAE development**. In EAE, splenic MDSCs promote Th17 cell differentiation and IL-17A secretion via IL-1β production in the presence of IL-6 and TGF-β1, which requires IL-1 receptors on Th17 cells. iNKT cells promote splenic MDSC expansion via GM-CSF and enhance their suppressive activities via IFN-γ and IL-4. Splenic M-MDSCs inhibit the proliferation of CD8 and CD4 T cells and induce apoptosis of CD4 T cells via NO production, whereas splenic PMN-MDSCs inhibit CD4 T cell proliferation via Arg-1 in a PD-L1/PD-1-dependent manner. IFN-γ in the microenvironment promotes PD-L1 expression on splenic PMN-MDSCs. Lung PMN-MDSCs reduce CD8 T cell proliferation via NO synthesis. PMN-MDSCs in the lung promote Th17 cell differentiation via IL-6 production in the presence of TGF-β1 and activated CD4 T cell-derived TNF-α, further contributing to MDSC production of IL-6. Abbreviations: iNKT cell: invariant natural killer T cell; M-MDSC: monocytic myeloid-derived suppressor cell; PMN-MDSC: polymorphonuclear myeloid-derived suppressor cell; Th17: T helper 17.

It is likely that MDSCs typically mediate EAE amelioration due to their well-known suppressive functions. However, as discussed below, this is not always the case. Intriguingly, MDSCs have been reported to enhance EAE development by promoting Th17 cell differentiation (Fig. 3) [45, 49]. Splenic MDSCs promoted Th17 cell differentiation in the presence of TGF-β, IL-6, and IL-1β *ex vivo*. Depletion of MDSCs with gemcitabine reduced the Th17 cell population *in vivo* and ameliorated EAE [45]. Consistently, a vigorous expansion of PMN-MDSCs in the lungs of EAE mice has been reported, which promoted Th17 cell polarization and IL-17A secretion via IL-6 production in the presence of TGF-β. In turn, TNF-α derived from activated CD4^+^ T cells contributed to MDSC-derived IL-6 production [49]. These findings provide new insights into the pleiotropic function of MDSCs and might help explain the failure of employing immunosuppressive MDSCs to control Th17/IL-17-dependent EAE development [45].

Recent evidence in cancer studies has indicated that M-MDSCs can differentiate into mature myeloid cells such as monocyte-derived macrophages and dendritic cells, while PMN-MDSCs are considered terminal differentiated cells [61, 62]. Notably, neutrophils can differentiate into PMN-MDSCs in EAE mice, although the precise mechanisms remain unclear [42]. Consistent with their dual roles discussed above, the maturation of MDSCs can either ameliorate or exacerbate EAE [52, 63], yet further studies are required to elucidate the maturation process of MDSCs in EAE.

## 5. Ly6C^high^ myeloid cells in EAE

In the context of EAE studies, there exist cell types that exhibit the same surface markers as MDSC and acquire MDSC-like properties, yet are not explicitly referred to as MDSCs. For instance, splenic CD11b^+^ Ly6C^high^ monocytes suppressed T cell proliferation and induced T cell apoptosis during EAE [48]. Additionally, Ly6C^high^ myeloid cells traffick to the CNS in response to CCR2 and exhibit significant plasticity in EAE development [64-67]. These cells within the CNS undergo progressive activation during EAE development, transiting their function from antigen presentation to MDSC-like T cell suppression [67]. Indeed, studies have revealed the high plasticity of CNS myeloid cells in the EAE setting and noticed their phenotype and functional evolution during the disease course [68, 69]. These findings might question the dichotomous role of MDSCs observed in EAE studies as labeling Ly6C^high^ cells as MDSCs without considering their specific functions that may have contributed to inconsistent results in studies, emphasizing the need for further investigation and clarification.

## 6. Potential therapeutic effects of MDSCs in MS/EAE

Autoimmune disorders, including MS, are often treated with immunosuppressive drugs that are considered the treatment gold standard and highly effective. However, long-term administration of high doses of these drugs may make patients more prone to life-threatening opportunistic infections and malignancies. In this regard, the use of *ex vivo* generated MDSCs and *in vivo* induction of MDSCs as immunotherapeutics are more reasonable and attractive approaches for MS patients.

### 6.1 Adoptive transfer of MDSCs

Several studies have provided insights into the direct use of MDSCs for EAE treatment. Adoptive transfer of MDSCs, especially PMN-MDSCs, to EAE mice has shown promising results. For instance, intravenous infusion of transduced MOG-expressing MDSCs before EAE induction protected against EAE by reducing activated CD4^+^ T cells and increasing B cells with a regulatory phenotype [70]. Additionally, pre- and post-treatment with PMN-MDSCs generated from healthy C57BL/6 mice prevented EAE progression and reduced the frequency of Th17 cells [71]. Moreover, adoptive transfer of PMN-MDSCs from MOG-immunized mice ameliorated EAE, reduced demyelination, and delayed disease onset [44]. Interestingly, after adoptive transfer of MDSCs activated with prostaglandin E2, immunized dark agouti rats showed ameliorated EAE symptoms, which were associated with the prevention of gut barrier disruption and preservation of gut microbial diversity [72]. However, specific interactions between MDSCs and microbiota remain poorly understood and require further investigation.

### 6.2 Therapeutic expansion of MDSCs in EAE

EAE attenuation facilitated by some treatments has been linked to MDSC expansion. For instance, oral administration of cannabidiol (CBD), a plant-derived bioactive compound with potent anti-inflammatory properties, attenuated EAE progression with an expansion of suppressive M-MDSCs in the spleen and PMN-MDSCs in the CNS [73]. Likewise, cannabinoid-mediated up-regulation of M-MDSCs was also noticed in another animal model of MS-Theiler’s virus-induced demyelination disease [74]. Not surprisingly, MDSC depletion eliminated the beneficial effects offered by CBD treatment, and adoptive transfer of CBD-induced MDSCs attenuated established EAE [75]. Interestingly, another study showed MDSC-independent protective effects of CBD treatment and, instead, suppression of EAE was attributed to an early inhibition of splenic IFN-γ^+^ CD8^+^ T cells [76].

In addition, expansion of MDSCs was reported in EAE mice treated with NAD, IFN-β, or SNJ-1945 (a calpain inhibitor) [77-79]. Significantly, the abundance of M-MDSCs has been identified as a crucial and specific biomarker to evaluate the clinical effectiveness and responsiveness of fingolimod treatment in EAE mice [43]. Meanwhile, the selective depletion of endogenously activated protein C (an anticoagulant involved in cross-talk between the coagulation and immune systems) [80], prolonged residence of an albumin-IL-4 fusion protein in secondary lymphoid [81], genetic deficiency of MiR-223 (a key mediator of myeloid cell development and function) were associated with MDSC expansion and EAE amelioration [40]. However, the precise causal relationship between MDSC expansion and treatment-mediated EAE amelioration remains elusive. Whether these treatments directly expand MDSCs and then alleviate EAE or whether the expanded MDSCs are the result of EAE amelioration remains to be uncovered. Further studies are needed to address this relationship and the underlying mechanism of MDSC expansion. Thus, while naturally occurring MDSCs may not be sufficient to control EAE, their therapeutic expansion might have some beneficial effects in limiting EAE development [12].

## 7. Conclusions

Since the frequency and numbers of M-MDSCs and PMN-MDSCs show variations in RRMS patients, using them as novel biomarkers for MS disease prediction and treatment responses has received substantial attention and shows significant potential. Few studies have investigated the functional roles of MDSCs in MS, but the mouse EAE model has revealed the functional roles of MDSCs in CNS inflammation, providing an opportunity to explore MDSC-based cell therapies for MS. It is reported that MDSCs play a dual role in EAE development. The beneficial effects provided by MDSCs in EAE are achieved through various mechanisms including suppressing autoreactive T cells, inducing Tregs, promoting remyelination, preventing accumulation of pathogenic B cells, and interacting with iNKT cells. Many studies have highlighted an association between MDSC expansion and EAE amelioration. Adoptive transfer of MDSCs to MOG-immunized mice reduced disease severity, suggesting a promising opportunity to treat MS with MDSC-based immunotherapy. However, the suppressive role of M-MDSCs in MS/EAE remains poorly understood, and the MDSC subset that may provide superior immunotherapeutic activities remains to be identified. The deleterious effects offered by MDSCs in EAE development are mainly attributed to their ability to promote Th17 cell differentiation. Further, whether M-MDSCs contribute to these effects is unclear. Therefore, developing MDSC-based immunotherapies will require further knowledge of their phenotype, differentiation, and cellular functions in MS/EAE [8].

Studies on the role of MDSCs in other autoimmune diseases might provide deeper insight into their potential therapeutic effects. Similar to the results obtained in MS and EAE, conflicting data exist on the role of MDSCs in RA and SLE. Studies have shown that the adoptive transfer of PMN-MDSCs from collagen-induced arthritis mice attenuates the severity of joint inflammation [82], and intravenous infusion of MDSCs attenuates autoimmunity in the sanroque mouse model of SLE [59]. However, other studies have revealed contrasting findings that MDSCs in arthritic or lupus mice promoted Th17 cell polarization [83, 84]. MDSC depletion suppressed T cell proliferation in collagen-induced arthritis mice and adoptive transfer of MDSCs restored Th17 differentiation and disease severity [83]. These divergent findings suggest pleiotropic functions of MDSCs in different physiological and pathological conditions. Since MDSCs have a dual role in EAE and other autoimmune diseases, employing these cells for immunotherapies is associated with substantial risks. Overall, developing effective MDSC-based immunotherapies for MS/EAE will require further studies to understand the molecular mechanisms underlying the protective or deleterious role of distinct MDSC subsets in MS/EAE.

## 8. Outstanding questions

MDSCs have been well-explored in cancer studies, but few studies on MS patients and EAE have been performed. To enhance our understanding of MDSCs in MS and to apply these insights to disease prevention and treatment, several questions will need to be addressed.

At present, distinguishing M-MDSCs and PMN-MDSCs from monocytes and neutrophils in humans and mice remains challenging. MDSCs are a heterogeneous population often defined by distinct markers, resulting in inconsistent results between different studies. Therefore, the definition of distinct MDSC subsets needs to be standardized.

An important research question pertains to the origin of these expanded MDSCs in EAE/MS. Whether these MDSCs were converted from differentiated monocytes and neutrophils or were a consequence of the affected precursor cell differentiation warrants further investigation [61].

Furthermore, while existing data suggest significant promise for adoptive transfer of MDSCs in EAE amelioration, safety concerns of MDSC-based cell therapies remain, as in some experimental conditions MDSCs can promote pathogenic Th17 cell differentiation in the EAE model. The underlying molecular mechanisms of their dual roles in EAE require further investigation.

Moreover, realizing the full potential of MDSCs as MS disease biomarkers and therapeutics will require a better understanding of the complex interactions between MDSCs and other disease-related cellular subsets.

## References

[1] Thompson AJ, Baranzini SE, Geurts J, Hemmer B, Ciccarelli O (2018). Multiple sclerosis. Lancet, 391:1622-1636. 29576504 10.1016/S0140-6736(18)30481-1

[2] Olsson T, Barcellos LF, Alfredsson L (2017). Interactions between genetic, lifestyle and environmental risk factors for multiple sclerosis. Nat Rev Neurol, 13:25-36. 27934854 10.1038/nrneurol.2016.187

[3] Codarri L, Greter M, Becher B (2013). Communication between pathogenic T cells and myeloid cells in neuroinflammatory disease. Trends Immunol, 34:114-119. 23116549 10.1016/j.it.2012.09.007

[4] Van Kaer L, Postoak JL, Wang C, Yang G, Wu L (2019). Innate, innate-like and adaptive lymphocytes in the pathogenesis of MS and EAE. Cell Mol Immunol, 16:531-539. 30874627 10.1038/s41423-019-0221-5 PMC6804597

[5] Kesselring J, Beer S (2005). Symptomatic therapy and neurorehabilitation in multiple sclerosis. Lancet Neurol, 4:643-652. 16168933 10.1016/S1474-4422(05)70193-9

[6] Goverman J (2009). Autoimmune T cell responses in the central nervous system. Nat Rev Immunol, 9:393-407. 19444307 10.1038/nri2550 PMC2813731

[7] Gabrilovich DI, Nagaraj S (2009). Myeloid-derived suppressor cells as regulators of the immune system. Nat Rev Immunol, 9:162-174. 19197294 10.1038/nri2506 PMC2828349

[8] Crook KR, Liu P (2014). Role of myeloid-derived suppressor cells in autoimmune disease. World J Immunol, 4:26. 25621222 10.5411/wji.v4.i1.26 PMC4302755

[9] Jiménez-Cortegana C, Galassi C, Klapp V, Gabrilovich DI, Galluzzi L (2022). Myeloid-derived suppressor cells and radiotherapy. Cancer Immunol Res, 10:545-557. 35426936 10.1158/2326-6066.CIR-21-1105

[10] Nagaraj S, Collazo M, Corzo CA, Youn J-I, Ortiz M, Quiceno D, et al. (2009). Regulatory myeloid suppressor cells in health and disease. Cancer Res, 69:7503-7506. 19752086 10.1158/0008-5472.CAN-09-2152 PMC2756310

[11] Talmadge JE, Gabrilovich DI (2013). History of myeloid-derived suppressor cells. Nat Rev Cancer, 13:739-752. 24060865 10.1038/nrc3581 PMC4358792

[12] Veglia F, Sanseviero E, Gabrilovich DI (2021). Myeloid-derived suppressor cells in the era of increasing myeloid cell diversity. Nat Rev Immunol, 21:485-498. 33526920 10.1038/s41577-020-00490-y PMC7849958

[13] Bronte V, Brandau S, Chen S-H, Colombo MP, Frey AB, Greten TF, et al. (2016). Recommendations for myeloid-derived suppressor cell nomenclature and characterization standards. Nat Commun, 7:1-10. 10.1038/ncomms12150 PMC4935811 27381735

[14] Gabrilovich DI (2017). Myeloid-Derived Suppressor Cells. Cancer Immunol Res, 5:3-8. 28052991 10.1158/2326-6066.CIR-16-0297 PMC5426480

[15] Condamine T, Dominguez GA, Youn J-I, Kossenkov AV, Mony S, Alicea-Torres K, et al. (2016). Lectin-type oxidized LDL receptor-1 distinguishes population of human polymorphonuclear myeloid-derived suppressor cells in cancer patients. Sci Immunol, 1:aaf8943-aaf8943. 28417112 10.1126/sciimmunol.aaf8943 PMC5391495

[16] Mastio J, Condamine T, Dominguez G, Kossenkov AV, Donthireddy L, Veglia F, et al. (2019). Identification of monocyte-like precursors of granulocytes in cancer as a mechanism for accumulation of PMN-MDSCs. J Exp Med, 216:2150-2169. 31239386 10.1084/jem.20181952 PMC6719429

[17] Alshetaiwi H, Pervolarakis N, McIntyre LL, Ma D, Nguyen Q, Rath JA, et al. (2020). Defining the emergence of myeloid-derived suppressor cells in breast cancer using single-cell transcriptomics. Sci Immunol, 5:eaay6017. 32086381 10.1126/sciimmunol.aay6017 PMC7219211

[18] Gabrilovich DI (2021). The dawn of myeloid-derived suppressor cells: identification of arginase I as the mechanism of immune suppression. Cancer Res, 81:3953-3955. 34341063 10.1158/0008-5472.CAN-21-1237

[19] Goldmann O, Beineke A, Medina E (2017). Identification of a novel subset of myeloid-derived suppressor cells during chronic staphylococcal infection that resembles immature eosinophils. J Infect Dis, 216:1444-1451. 29029332 10.1093/infdis/jix494

[20] Gantt S, Gervassi A, Jaspan H, Horton H (2014). The Role of Myeloid-Derived Suppressor Cells in Immune Ontogeny. Front Immunol, 5:387. 25165466 10.3389/fimmu.2014.00387 PMC4131407

[21] Kropf P, Baud D, Marshall SE, Munder M, Mosley A, Fuentes JM, et al. (2007). Arginase activity mediates reversible T cell hyporesponsiveness in human pregnancy. Eur J Immunol, 37:935-945. 17330821 10.1002/eji.200636542 PMC2699382

[22] Rieber N, Gille C, Köstlin N, Schäfer I, Spring B, Ost M, et al. (2013). Neutrophilic myeloid-derived suppressor cells in cord blood modulate innate and adaptive immune responses. Clin Exp Immunol, 174:45-52. 23701226 10.1111/cei.12143 PMC3784212

[23] Budhwar S, Verma P, Verma R, Rai S, Singh K (2018). The Yin and Yang of Myeloid Derived Suppressor Cells. Front Immunol, 9. 30555467 10.3389/fimmu.2018.02776 PMC6280921

[24] Almand B, Clark JI, Nikitina E, van Beynen J, English NR, Knight SC, et al. (2001). Increased production of immature myeloid cells in cancer patients: a mechanism of immunosuppression in cancer. J Immunol, 166:678-689. 11123353 10.4049/jimmunol.166.1.678

[25] Köstlin N, Schoetensack C, Schwarz J, Spring B, Marmé A, Goelz R, et al. (2018). Granulocytic myeloid-derived suppressor cells (GR-MDSC) in breast milk (BM); GR-MDSC accumulate in human BM and modulate T-cell and monocyte function. Front Immunol, 9:1098. 29868036 10.3389/fimmu.2018.01098 PMC5966528

[26] Yaseen MM, Abuharfeil NM, Darmani H, Daoud A (2021). Recent advances in myeloid-derived suppressor cell biology. Front Med, 15:232-251. 32876877 10.1007/s11684-020-0797-2

[27] Rodriguez PC, Quiceno DG, Ochoa AC (2007). L-arginine availability regulates T-lymphocyte cell-cycle progression. Blood, 109:1568-1573. 17023580 10.1182/blood-2006-06-031856 PMC1794048

[28] Rodriguez PC, Quiceno DG, Zabaleta J, Ortiz B, Zea AH, Piazuelo MB, et al. (2004). Arginase I production in the tumor microenvironment by mature myeloid cells inhibits T-cell receptor expression and antigen-specific T-cell responses. Cancer Res, 64:5839-5849. 15313928 10.1158/0008-5472.CAN-04-0465

[29] Kwak Y, Kim HE, Park SG (2015). Insights into Myeloid-Derived Suppressor Cells in Inflammatory Diseases. Arch Immunol Ther Exp (Warsz), 63:269-285. 25990434 10.1007/s00005-015-0342-1

[30] Kusmartsev S, Gabrilovich DI (2003). Inhibition of myeloid cell differentiation in cancer: the role of reactive oxygen species. J Leukoc Biol, 74:186-196. 12885935 10.1189/jlb.0103010

[31] Brito C, Naviliat M, Tiscornia AC, Vuillier F, Gualco G, Dighiero G, et al. (1999). Peroxynitrite inhibits T lymphocyte activation and proliferation by promoting impairment of tyrosine phosphorylation and peroxynitrite-driven apoptotic death. J Immunol, 162:3356-3366. 10092790

[32] Nagaraj S, Gupta K, Pisarev V, Kinarsky L, Sherman S, Kang L, et al. (2007). Altered recognition of antigen is a mechanism of CD8+ T cell tolerance in cancer. Nat Med, 13:828-835. 17603493 10.1038/nm1609 PMC2135607

[33] Huang B, Pan P-Y, Li Q, Sato AI, Levy DE, Bromberg J, et al. (2006). Gr-1+ CD115+ immature myeloid suppressor cells mediate the development of tumor-induced T regulatory cells and T-cell anergy in tumor-bearing host. Cancer Res, 66:1123-1131. 16424049 10.1158/0008-5472.CAN-05-1299

[34] Movahedi K, Guilliams M, Van den Bossche J, Van den Bergh R, Gysemans C, Beschin A, et al. (2008). Identification of discrete tumor-induced myeloid-derived suppressor cell subpopulations with distinct T cell-suppressive activity. Blood, 111:4233-4244. 18272812 10.1182/blood-2007-07-099226

[35] Dugast A-S, Haudebourg T, Coulon F, Heslan M, Haspot F, Poirier N, et al. (2008). Myeloid-derived suppressor cells accumulate in kidney allograft tolerance and specifically suppress effector T cell expansion. J Immunol, 180:7898-7906. 18523253 10.4049/jimmunol.180.12.7898

[36] Li H, Han Y, Guo Q, Zhang M, Cao X (2009). Cancer-expanded myeloid-derived suppressor cells induce anergy of NK cells through membrane-bound TGF-β1. J Immunol, 182:240-249. 19109155 10.4049/jimmunol.182.1.240

[37] Nausch N, Galani IE, Schlecker E, Cerwenka A (2008). Mononuclear myeloid-derived “suppressor” cells express RAE-1 and activate natural killer cells. Blood, 112:4080-4089. 18753637 10.1182/blood-2008-03-143776 PMC2582006

[38] Iacobaeus E, Douagi I, Jitschin R, Marcusson-Stahl M, Andren AT, Gavin C, et al. (2018). Phenotypic and functional alterations of myeloid-derived suppressor cells during the disease course of multiple sclerosis. Immunol Cell Biol, 96:820-830. 29569304 10.1111/imcb.12042

[39] D'Amico E, Zanghi A, Parrinello NL, Romano A, Palumbo GA, Chisari CG, et al. (2022). Immunological Subsets Characterization in Newly Diagnosed Relapsing-Remitting Multiple Sclerosis. Front Immunol, 13:819136. 35273601 10.3389/fimmu.2022.819136 PMC8902351

[40] Cantoni C, Cignarella F, Ghezzi L, Mikesell B, Bollman B, Berrien-Elliott MM, et al. (2017). Mir-223 regulates the number and function of myeloid-derived suppressor cells in multiple sclerosis and experimental autoimmune encephalomyelitis. Acta Neuropathol, 133:61-77. 27704281 10.1007/s00401-016-1621-6 PMC5423756

[41] Wang Z, Zheng G, Li G, Wang M, Ma Z, Li H, et al. (2020). Methylprednisolone alleviates multiple sclerosis by expanding myeloid-derived suppressor cells via glucocorticoid receptor beta and S100A8/9 up-regulation. J Cell Mol Med, 24:13703-13714. 33094923 10.1111/jcmm.15928 PMC7753844

[42] Knier B, Hiltensperger M, Sie C, Aly L, Lepennetier G, Engleitner T, et al. (2018). Myeloid-derived suppressor cells control B cell accumulation in the central nervous system during autoimmunity. Nat Immunol, 19:1341-1351. 30374128 10.1038/s41590-018-0237-5 PMC6241855

[43] Camacho-Toledano C, Machín-Díaz I, Calahorra L, Cabañas-Cotillas M, Otaegui D, Castillo-Triviño T, et al. (2022). Peripheral Myeloid-Derived Suppressor Cells are good biomarkers of the efficacy of Fingolimod in Multiple Sclerosis. J Neuroinflammation, 19:1-22. 36403026 10.1186/s12974-022-02635-3 PMC9675277

[44] Ioannou M, Alissafi T, Lazaridis I, Deraos G, Matsoukas J, Gravanis A, et al. (2012). Crucial role of granulocytic myeloid-derived suppressor cells in the regulation of central nervous system autoimmune disease. J Immunol, 188:1136-1146. 22210912 10.4049/jimmunol.1101816

[45] Yi H, Guo C, Yu X, Zuo D, Wang XY (2012). Mouse CD11b+Gr-1+ myeloid cells can promote Th17 cell differentiation and experimental autoimmune encephalomyelitis. J Immunol, 189:4295-4304. 23034169 10.4049/jimmunol.1200086 PMC3478426

[46] Moline-Velazquez V, Cuervo H, Vila-Del Sol V, Ortega MC, Clemente D, de Castro F (2011). Myeloid-derived suppressor cells limit the inflammation by promoting T lymphocyte apoptosis in the spinal cord of a murine model of multiple sclerosis. Brain Pathol, 21:678-691. 21507122 10.1111/j.1750-3639.2011.00495.x PMC8094047

[47] Melero-Jerez C, Alonso-Gomez A, Monivas E, Lebron-Galan R, Machin-Diaz I, de Castro F, et al. (2020). The proportion of myeloid-derived suppressor cells in the spleen is related to the severity of the clinical course and tissue damage extent in a murine model of multiple sclerosis. Neurobiol Dis, 140:104869. 32278882 10.1016/j.nbd.2020.104869

[48] Zhu B, Bando Y, Xiao S, Yang K, Anderson AC, Kuchroo VK, et al. (2007). CD11b+ Ly-6Chi suppressive monocytes in experimental autoimmune encephalomyelitis. J Immunol, 179:5228-5237. 17911608 10.4049/jimmunol.179.8.5228

[49] Glenn JD, Liu C, Whartenby KA (2019). Frontline Science: Induction of experimental autoimmune encephalomyelitis mobilizes Th17-promoting myeloid derived suppressor cells to the lung. J Leukoc Biol, 105:829-841. 30762897 10.1002/JLB.4HI0818-335R

[50] Glenn JD, Smith MD, Xue P, Chan-Li Y, Collins S, Calabresi PA, et al. (2017). CNS-targeted autoimmunity leads to increased influenza mortality in mice. J Exp Med, 214:297-307. 28057805 10.1084/jem.20160517 PMC5294848

[51] Wegner A, Verhagen J, Wraith DC (2017). Myeloid-derived suppressor cells mediate tolerance induction in autoimmune disease. Immunology, 151:26-42. 28140447 10.1111/imm.12718 PMC5382345

[52] Moline-Velazquez V, Ortega MC, Vila del Sol V, Melero-Jerez C, de Castro F, Clemente D (2014). The synthetic retinoid Am80 delays recovery in a model of multiple sclerosis by modulating myeloid-derived suppressor cell fate and viability. Neurobiol Dis, 67:149-164. 24709559 10.1016/j.nbd.2014.03.017

[53] Tanwar S, Oguz C, Metidji A, Dahlstrom E, Barbian K, Kanakabandi K, et al. (2020). Type I IFN signaling in T regulatory cells modulates chemokine production and myeloid derived suppressor cells trafficking during EAE. J Autoimmun, 115:102525. 32709481 10.1016/j.jaut.2020.102525 PMC7712497

[54] Melero-Jerez C, Fernandez-Gomez B, Lebron-Galan R, Ortega MC, Sanchez-de Lara I, Ojalvo AC, et al. (2021). Myeloid-derived suppressor cells support remyelination in a murine model of multiple sclerosis by promoting oligodendrocyte precursor cell survival, proliferation, and differentiation. Glia, 69:905-924. 33217041 10.1002/glia.23936 PMC7894183

[55] Lelis FJ, Jaufmann J, Singh A, Fromm K, Teschner AC, Pöschel S, et al. (2017). Myeloid-derived suppressor cells modulate B-cell responses. Immunol Lett, 188:108-115. 28687234 10.1016/j.imlet.2017.07.003

[56] Li Y, Tu Z, Qian S, Fung JJ, Markowitz SD, Kusner LL, et al. (2014). Myeloid-derived suppressor cells as a potential therapy for experimental autoimmune myasthenia gravis. J Immunol, 193:2127-2134. 25057008 10.4049/jimmunol.1400857 PMC4784709

[57] Wang C, Zhang N, Qi L, Yuan J, Wang K, Wang K, et al. (2017). Myeloid-derived suppressor cells inhibit T follicular helper cell immune response in Japanese encephalitis virus infection. J Immunol, 199:3094-3105. 28978693 10.4049/jimmunol.1700671

[58] Xu X, Meng Q, Erben U, Wang P, Glauben R, Kühl AA, et al. (2017). Myeloid-derived suppressor cells promote B-cell production of IgA in a TNFR2-dependent manner. Cell Mol Immunol, 14:597-606. 27133471 10.1038/cmi.2015.103 PMC5520412

[59] Park MJ, Lee SH, Kim EK, Lee EJ, Park SH, Kwok SK, et al. (2016). Myeloid-derived suppressor cells induce the expansion of regulatory B cells and ameliorate autoimmunity in the sanroque mouse model of systemic lupus erythematosus. Arthritis Rheumatol, 68:2717-2727. 27214349 10.1002/art.39767

[60] Parekh VV, Wu L, Olivares-Villagomez D, Wilson KT, Van Kaer L (2013). Activated invariant NKT cells control central nervous system autoimmunity in a mechanism that involves myeloid-derived suppressor cells. J Immunol, 190:1948-1960. 23345328 10.4049/jimmunol.1201718 PMC3577977

[61] Tcyganov E, Mastio J, Chen E, Gabrilovich DI (2018). Plasticity of myeloid-derived suppressor cells in cancer. Curr Opin Immunol, 51:76-82. 29547768 10.1016/j.coi.2018.03.009 PMC5943174

[62] Veglia F, Perego M, Gabrilovich D (2018). Myeloid-derived suppressor cells coming of age. Nat Immunol, 19:108-119. 29348500 10.1038/s41590-017-0022-x PMC5854158

[63] Dagkonaki A, Papalambrou A, Avloniti M, Gkika A, Evangelidou M, Androutsou M-E, et al. (2022). Maturation of circulating Ly6ChiCCR2+ monocytes by mannan-MOG induces antigen-specific tolerance and reverses autoimmune encephalomyelitis. Front Immunol, 13. 10.3389/fimmu.2022.972003 PMC9501702 36159850

[64] King IL, Dickendesher TL, Segal BM (2009). Circulating Ly-6C+ myeloid precursors migrate to the CNS and play a pathogenic role during autoimmune demyelinating disease. Blood, 113:3190-3197. 19196868 10.1182/blood-2008-07-168575 PMC2665891

[65] Mildner A, Mack M, Schmidt H, Brück W, Djukic M, Zabel MD, et al. (2009). CCR2+ Ly-6Chi monocytes are crucial for the effector phase of autoimmunity in the central nervous system. Brain, 132:2487-2500. 19531531 10.1093/brain/awp144

[66] Saederup N, Cardona AE, Croft K, Mizutani M, Cotleur AC, Tsou C-L, et al. (2010). Selective chemokine receptor usage by central nervous system myeloid cells in CCR2-red fluorescent protein knock-in mice. PloS one, 5:e13693. 21060874 10.1371/journal.pone.0013693 PMC2965160

[67] Zhu B, Kennedy JK, Wang Y, Sandoval-Garcia C, Cao L, Xiao S, et al. (2011). Plasticity of Ly-6Chi myeloid cells in T cell regulation. J Immunol, 187:2418-2432. 21824867 10.4049/jimmunol.1100403 PMC3159773

[68] Locatelli G, Theodorou D, Kendirli A, Jordao MJC, Staszewski O, Phulphagar K, et al. (2018). Mononuclear phagocytes locally specify and adapt their phenotype in a multiple sclerosis model. Nat Neurosci, 21:1196-1208. 30127427 10.1038/s41593-018-0212-3

[69] Giles DA, Washnock-Schmid JM, Duncker PC, Dahlawi S, Ponath G, Pitt D, et al. (2018). Myeloid cell plasticity in the evolution of central nervous system autoimmunity. Ann Neurol, 83:131-141. 29283442 10.1002/ana.25128 PMC5876132

[70] Casacuberta-Serra S, Costa C, Eixarch H, Mansilla MJ, Lopez-Estevez S, Martorell L, et al. (2016). Myeloid-derived suppressor cells expressing a self-antigen ameliorate experimental autoimmune encephalomyelitis. Exp Neurol, 286:50-60. 27693617 10.1016/j.expneurol.2016.09.012

[71] Ghorbani MM, Farazmandfar T, Abediankenari S, Hassannia H, Maleki Z, Shahbazi M (2022). Treatment of EAE mice with Treg, G-MDSC and IL-2: a new insight into cell therapy for multiple sclerosis. Immunotherapy, 14:789-798 35678041 10.2217/imt-2021-0045

[72] Radojević D, Bekić M, Gruden-Movsesijan A, Ilić N, Dinić M, Bisenić A, et al. (2022). Myeloid-derived suppressor cells prevent disruption of the gut barrier, preserve microbiota composition, and potentiate immunoregulatory pathways in a rat model of experimental autoimmune encephalomyelitis. Gut microbes, 14:2127455. 36184742 10.1080/19490976.2022.2127455 PMC9543149

[73] Dopkins N, Miranda K, Wilson K, Holloman BL, Nagarkatti P, Nagarkatti M (2022). Effects of Orally Administered Cannabidiol on Neuroinflammation and Intestinal Inflammation in the Attenuation of Experimental Autoimmune Encephalomyelitis. J Neuroimmune Pharmacol, 17:15-32 34757526 10.1007/s11481-021-10023-6 PMC10207886

[74] Mecha M, Feliú A, Machín I, Cordero C, Carrillo-Salinas F, Mestre L, et al. (2018). 2-AG limits Theiler's virus induced acute neuroinflammation by modulating microglia and promoting MDSCs. Glia, 66:1447-1463. 29484707 10.1002/glia.23317

[75] Elliott DM, Singh N, Nagarkatti M, Nagarkatti PS (2018). Cannabidiol Attenuates Experimental Autoimmune Encephalomyelitis Model of Multiple Sclerosis Through Induction of Myeloid-Derived Suppressor Cells. Front Immunol, 9:1782. 30123217 10.3389/fimmu.2018.01782 PMC6085417

[76] Nichols JM, Kummari E, Sherman J, Yang EJ, Dhital S, Gilfeather C, et al. (2021). CBD Suppression of EAE Is Correlated with Early Inhibition of Splenic IFN-gamma + CD8+ T Cells and Modest Inhibition of Neuroinflammation. J Neuroimmune Pharmacol, 16:346-362. 32440886 10.1007/s11481-020-09917-8 PMC7679272

[77] Wang JL, Li B, Tan GJ, Gai XL, Xing JN, Wang JQ, et al. (2020). NAD+ attenuates experimental autoimmune encephalomyelitis through induction of CD11b+ gr-1+ myeloid-derived suppressor cells. Biosci Rep, 40. 10.1042/BSR20200353 PMC7182659 32301489

[78] Melero-Jerez C, Suardiaz M, Lebron-Galan R, Marin-Banasco C, Oliver-Martos B, Machin-Diaz I, et al. (2019). The presence and suppressive activity of myeloid-derived suppressor cells are potentiated after interferon-beta treatment in a murine model of multiple sclerosis. Neurobiol Dis, 127:13-31. 30798007 10.1016/j.nbd.2019.02.014

[79] Trager N, Smith A, Wallace Iv G, Azuma M, Inoue J, Beeson C, et al. (2014). Effects of a novel orally administered calpain inhibitor SNJ-1945 on immunomodulation and neurodegeneration in a murine model of multiple sclerosis. J Neurochem, 130:268-279. 24447070 10.1111/jnc.12659 PMC4107076

[80] Alabanza LM, Esmon NL, Esmon CT, Bynoe MS (2013). Inhibition of endogenous activated protein C attenuates experimental autoimmune encephalomyelitis by inducing myeloid-derived suppressor cells. J Immunol, 191:3764-3777. 23997223 10.4049/jimmunol.1202556 PMC3800123

[81] Ishihara A, Ishihara J, Watkins EA, Tremain AC, Nguyen M, Solanki A, et al. (2021). Prolonged residence of an albumin-IL-4 fusion protein in secondary lymphoid organs ameliorates experimental autoimmune encephalomyelitis. Nat Biomed Eng, 5:387-398. 33046864 10.1038/s41551-020-00627-3

[82] Wang W, Jiao Z, Duan T, Liu M, Zhu B, Zhang Y, et al. (2015). Functional characterization of myeloid-derived suppressor cell subpopulations during the development of experimental arthritis. Eur J Immunol, 45:464-473. 25352399 10.1002/eji.201444799

[83] Zhang H, Wang S, Huang Y, Wang H, Zhao J, Gaskin F, et al. (2015). Myeloid-derived suppressor cells are proinflammatory and regulate collagen-induced arthritis through manipulating Th17 cell differentiation. Clin Immunol, 157:175-186. 25680967 10.1016/j.clim.2015.02.001 PMC4657752

[84] Ji J, Xu J, Zhao S, Liu F, Qi J, Song Y, et al. (2016). Myeloid-derived suppressor cells contribute to systemic lupus erythaematosus by regulating differentiation of Th17 cells and Tregs. Clin Sci, 130:1453-1467. 10.1042/CS20160311 27231253

